# Coronary heart disease mortality in severe *vs.* non-severe familial hypercholesterolaemia in the Simon Broome Register

**DOI:** 10.1016/j.atherosclerosis.2018.11.014

**Published:** 2019-02

**Authors:** Steve E. Humphries, Jackie A. Cooper, Nigel Capps, Paul N. Durrington, Ben Jones, Ian F.W. McDowell, Handrean Soran, Andrew H.W. Neil, Simon Broome Familial Hyperlipidaemia Register Group

**Affiliations:** a Centre for Cardiovascular Genetics, Institute of Cardiovascular Science, University College London, University Street, London, WC1E 6JJ, UK; b Department of Clinical Biochemistry, The Shrewsbury and Telford Hospital NHS Trust, Princess Royal Hospital, Telford, UK; c Cardiovascular Research Group, School of Clinical and Laboratory Sciences, University of Manchester, Manchester, UK; d Section of Investigative Medicine, Imperial College London, London, UK; e Department of Medical Biochemistry and Immunology, University Hospital of Wales, Cardiff, UK; f University Department of Medicine, Manchester University Hospitals NHS Foundation Trust, Manchester, UK; g Wolfson College, University of Oxford, Oxford, UK

**Keywords:** Severe heterozygous familial hypercholesterolemia, Coronary mortality

## Abstract

**Background and aims:**

The International Atherosclerosis Society (IAS) has proposed that patients with “severe” FH (SFH) would warrant early and more aggressive cholesterol-lowering treatment such as with PCSK9 inhibitors. SFH is diagnosed if LDL-cholesterol (LDLC) > 10 mmol/L, or LDLC >8.0 mmol/L plus one high-risk feature, or LDLC >5 mmol/L plus two high-risk features. Here we compare CHD mortality in SFH and non-SFH (NSFH) patients in the UK prospective Simon Broome Register since 1991, when statin use became routine.

**Methods:**

2929 definite or possible PFH patients (51% women) aged 20–79 years were recruited from 21 UK lipid clinics and followed prospectively between 1992 and 2016. The excess CHD standardised mortality ratio (SMR) compared to the England and Wales population was calculated (with 95% confidence intervals).

**Results:**

1982 (67.7%) patients met the SFH definition. Compared to the non-SFH, significantly (*p* < 0.001) more SFH patients had diagnosed CHD at baseline (24.6% *vs.* 17.5%), were current smokers (21.9% vs 10.2%) and had a BMI > 30 kg/m^2^ (14.9% *vs.* 7.8%). The SMR for CHD mortality was significantly (*p* = 0.007) higher for SFH (220 (184–261) (34,134 person years, 129 deaths observed, *vs*. 59 expected) compared to NSFH of 144 (98–203) (15,432 person years, 32 observed *vs*. 22 expected). After adjustment for traditional risk factors, the Hazard Ratio for CHD mortality in SFH *vs*. NSFH was 1.22 (0.80–1.87) *p* = 0.36, indicating that the excess risk was largely accounted for by these factors.

**Conclusions:**

CHD mortality remains elevated in treated FH, especially for SFH, emphasising the importance of optimal lipid-lowering and management of other risk factors.

## Highlights

• Patients with IAS-defined “severe” FH (SFH) are at highest risk of future CHD.
• In the UK Simon Broome FH Register ∼70% meet the SFH criteria.
• Those with SFH have 64% higher CHD mortality than non SFH patients.
• This is explained by their higher classical risk factors including untreated LDL-C.

## Graphical abstract

**Figure:**
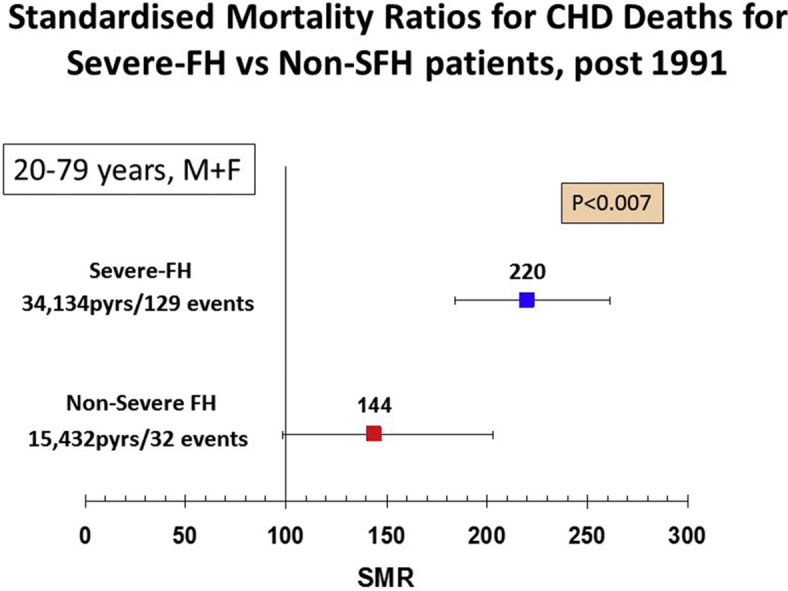

## 1 Introduction

Familial hypercholesterolaemia (FH) is a common autosomal dominant disorder caused by carriage of a mutation in one of several genes known to be involved in clearance of low-density lipoprotein cholesterol (LDL-C) particles from the blood [1,2]. The elevated levels of LDL-C from birth mean that patients with FH have a very high risk of developing coronary heart disease (CHD) at an early age [[3], [4], [5]]. In 2016, the International Atherosclerosis Society proposed that patients with “severe” FH (SFH) should be identified [6] since they might warrant early and more aggressive cholesterol-lowering treatment (e.g., with proprotein convertase subtilisin/kexin type 9 [PCSK9] inhibitors). SFH is diagnosed if the patient has LDL-cholesterol (LDL-C) >10 mmol/L or LDL-C >8.0 mmol/L plus one high-risk feature or LDL-C >5 mmol/L plus two high-risk features. High-risk features are age >40 years without treatment, smoking, male sex, lipoprotein(a) > 75 nmol/L, hypertension, diabetes mellitus, family history of early CHD in first-degree relatives, chronic kidney disease, and BMI >30 kg/m^2^ [6].

Here we compare the standardised mortality ratio (SMR) for CHD in SFH and non-SFH patients in the UK prospective Simon Broome Register, which has been following FH patients since 1988 for CHD and non-CHD causes of death [[7], [8], [9], [10]]. We use this data to estimate the SMR for CHD in SFH *vs*. Non-SFH (NSFH). The Simon Broome Registry categorises those with elevated LDL-C and early family history of heart disease and with tendon xanthomas as “definite FH (DFH)” and those with no tendon xanthomas as “possible FH (PFH)”, with CHD mortality being higher in DFH compared to PFH patients [[7], [8], [9], [10]], and we compare CHD mortality rates in those with SFH and DFH *vs*. PFH. We examine whether the CHD risk associated with SFH is similar in men and women, and we estimate the risk of CHD in SFH in different age groups, and to what extent the CHD SMR rates fall over time in SFH as potent statins become available. Finally, we estimate the extent to which the higher CHD mortality risk in SFH patients is explained by the included classical risk factors.

## 2 Materials and methods

The methods have been described previously [[7], [8], [9], [10]]. The characteristics of patients at registration were recorded on a standard registration form. A fasting venous blood specimen taken at the registration visit was used to determine serum total cholesterol, triglycerides, and high density lipoprotein, and was measured by the laboratories routinely used by the participating clinics. LDL-C concentrations were calculated using the Friedewald formula [11]. Registered patients were flagged by the National Health Service Central Registry and, in the event of death, a copy of the death certificate was provided. The underlying cause of death was coded by one investigator using the International Classification of Disease (ICD) 9th revision. All patients gave informed consent for inclusion in the Register. The study received approval from the local ethics committee of each participating centre. Patients were classified as having either Simon Broome (SB) definite FH or possible FH, using published criteria previously [[7], [8], [9], [10]], and by using the proposed severe FH criteria as described above and in Ref. [6]. Hypertension was defined as self-reported hypertension, and family history of early CHD in first-degree relatives was defined as recorded CHD under the age of 55 years in a male and 60 years in a female first-degree relative. Lipoprotein(a) and creatinine measures were not available in the majority of subjects so these variables were not included.

The analysis used a standard computer program for cohort studies [12]. Person-years of risk were aggregated into 5-year age groups and 5-year calendar periods and the expected number of deaths from specified causes were estimated. A total of 571 subjects were censored on reaching the age 80 years, and a further 50 patients who had emigrated were censored at the date of embarkation. The expected number of deaths from CHD (ICD codes 4100–4149) were calculated by applying the age and calendar-specific death rates for men and women in the general population of England and Wales to the person years (pyears) accumulated by men and women in the cohort. The standardised mortality ratio (SMR) was calculated from the ratio of the number of deaths observed to those expected, and was expressed as a percentage (SMR = 100 for the reference population), and the exact 95% confidence intervals were calculated. The test of significance used was a two-sided Poisson probability of observing the number of deaths that occurred given the expected number of deaths. A Cox-proportional hazard model was used to identify univariate baseline characteristics that were significantly associated with CHD mortality, and a stepwise model used to identify those that were independently associated using a significance level of 0.05 for entry to the model and 0.10 for elimination. A term for SFH/NSFH was forced into the model to examine whether the higher risk in SFH patients was explained by these factors. Main analyses were conducted on data obtained post 1991 in order to estimate CHD mortality in statin-treated patients. We also included the period 1980–91 in the analysis to examine the change in CHD mortality over time i.e. pre and post-statins. To do this, we divide the follow-up periods into that before January 1992, when statins were not routinely used, between January 1992–December 2008, during which time statin treatment became widely available, and from 2009 to December 2015, when it would be expected that FH patients would have their LDL-C levels managed with high potency statin treatment and or combination therapy with other lipid lowering agents such as ezetimibe.

## 3 Results

Of the 2929 registered patients with the required data, 201 (7.2%) had LDL-C >10.0 mmol/l, 423 (14.4%) had LDL-C below 10 mmol/l but above 8 mmol/l, and 1809 (61.8%) had LDL-C below 8 mmol/l but above 5.0 mmol/l (Supplementary Table 1), with 496 (16.9%) having LDL-C levels below 5.0 mmol/l. All of those with LDL> 10 mmol/l fulfil the SFH definition, while 96.6% (n = 409) of those with LDL-C <10 mmol/l but >8 mmol/l had one or more high risk factor and 75.8% (n = 1372) of those with LDL-C <8 mmol/l but >5.0 mmol/l had two or more high risk factors. In total, 1982 (67.7%) met the SFH definition. As shown in Table 1, compared to the NSFH group, significantly (all *p* < 0.001) more of those with SFH had diagnosed CHD (24.6% *vs*. 17.5%), were current smokers (21.9% *vs.* 10.2%) and had a BMI > 30 kg/m^2^ (14.9% *vs*. 7.8%). The prevalence of diabetes was low in both groups (1.0% *vs.* 0.6%). Compared to the NSFH patients, a significantly higher proportion of the SFH group had an SB clinical diagnosis of DFH (55.8% *vs.* 49.5% *p* = 0.02).

**Table 1:** Mean (SD) characteristics in those with severe-FH and non-severe-FH.

	Non-severe FH N = 947	Severe FH N = 1982	*p* value
Median age [IQR]	44.0 [28.8–57.4]	45.5 (34.5–56.3)	0.017
Previous MI (No (%))	77 (8.3%, n = 931)	220 (11.3%, n = 1943)	0.012
Current/past angina (No (%))	120 (13.0%, N = 923)	374 (19.8, N = 1889)	<0.001
Diagnosed CHD (No (%))	166 (17.5%, N = 947)	487 (24.6%, N = 1982)	<0.001
Previous stroke (No (%))	12 (1.3%, N = 945)	20 (1.0%, N = 1971)	0.536
Diagnosed diabetes (No (%))	6 (0.6%, N = 946)	20 (1.0%, N = 1976)	0.309
Current cigarette smoker (No (%))	94 (10.2%, N = 921)	424 (21.9%, N = 1937)	<0.001
Systolic BP (mmHg)	129.6 (20.6, N = 884)	133.3 (21.0, N = 1919)	<0.001
Diastolic BP (mmHg)	78.6 (11.1, N = 883)	80.5 (11.6, N = 1917)	<0.001
BMI (kg/m^2^)	24.4 (4.2, N = 830)	25.6 (4.4, N = 1762)	<0.001
BMI>30	65 (7.8%, N = 830)	264 (15.0%, N = 1762)	<0.001
Total cholesterol (mmol/l)	6.79 (1.41, N = 941)	8.39 (2.09, N = 1969)	<0.001
Triglyceride^a^ (mmol/l)	1.26 (0.8–1.9, N = 937)	1.45 (1–2.1, N = 1959)	<0.001
HDL-C (mmol/l)	1.41 (0.4, N = 930)	1.26 (0.35, N = 1910)	<0.001
LDL-C (mmol/l)	5.00 (1.43, N = 924)	6.71 (2.07, N = 1892)	<0.001
DFH (number (%))	469 (49.5%, N = 947)	1105 (55.8%, N = 1982)	0.002

Diagnosed CHD includes previous MI/angina, etc.

a Geom. mean [IQR]. DFH = Simon Broome clinical diagnosis of definite FH (ref).

**Fig. 1:**
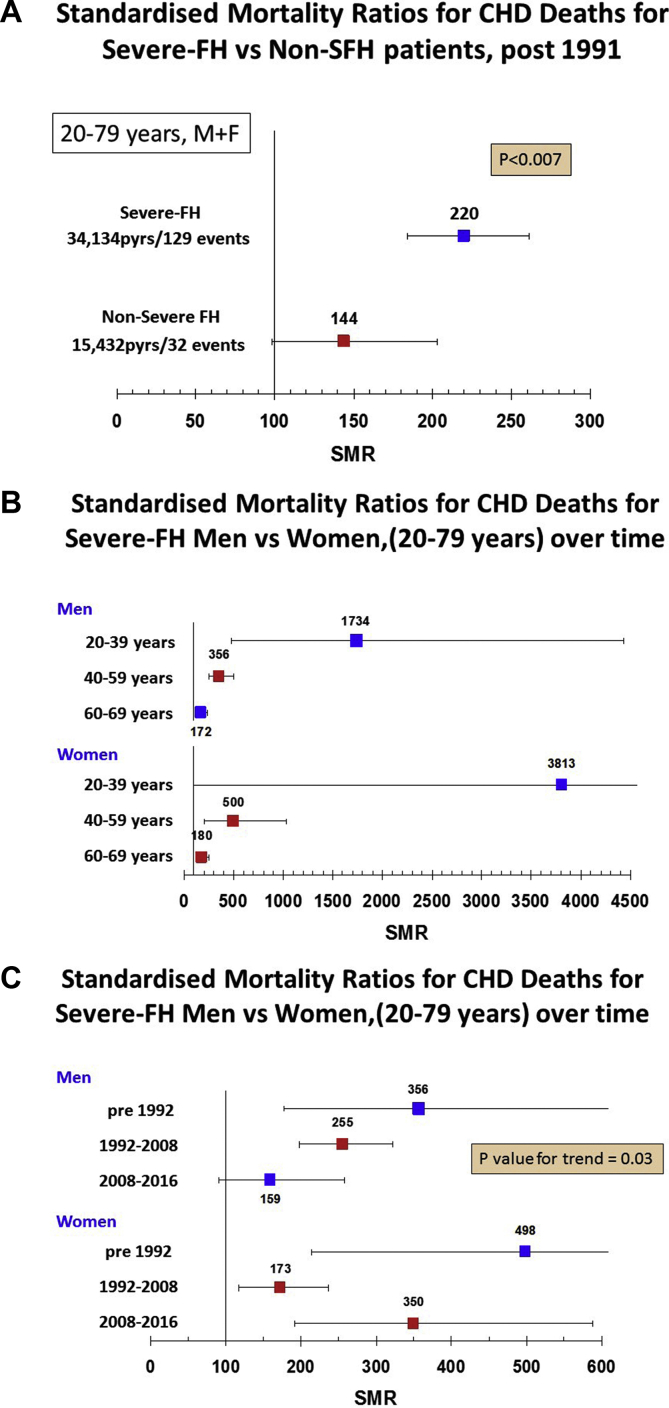
SMR CHD deaths. (A) For severe-FH and non-severe-FH patients post 1991, age 20–79 years. (B) For severe-FH male and female patients in different age catagories. (C) For severe-FH male and female patients age 20–79 years in different time periods.

Overall, there were 185 deaths from CHD. As shown in Fig. 1A, post 1991, in those with SFH, the SMR for CHD mortality was 64% higher (220 (184–261) compared to non-SFH of 144 (98–203) (*p* = 0.007). As shown in Supplementary Table 2, for those with SFH, the SMR for CHD mortality was significantly higher in those with a Simon Broome diagnosis of definite FH compared to those with possible FH (255 (202–318) *vs*. 179 (133–237), *p* = 0.05). By contrast, in those with non-SFH, there was no difference in the SMR in definite *vs*. possible FH patients (Supplementary Table 2). The overall ten-year CHD mortality rate (Kaplan-Meier estimate) for NSFH at baseline was 1.5% and for SFH was 3.5%. After adjusting for age, this equated to a rate of 0.5% at age 40, 1.3% at age 50 and 3.1% at age 60 for those with NSFH, with rates in those with SFH being 2.2%, 3.7% and 6.2% respectively.

A substantially elevated SMR for CHD in SFH was observed in both males (237 (192–290) and females (226 (169–296)), while in NSFH, the SMRs were lower but still statistically significantly higher in males (168 (106–251) but not in females (109 (56–190)) (Supplementary Fig. 1). We also estimated the CHD SMR in SFH males and females by age categories at registration, and data are presented in Fig. 1B. In both males and females in the age range 20–39 years, the SMR was extremely high but the number of observed events was very low and the confidence intervals large. In the 40–59 year age range, where numbers of observed deaths is higher, the SMR in males was 367 (263–501) (10,483 pyears; 40 deaths *vs*. 10.8 expected) and in females was 611v305-1093) (6194 pyears; 11 deaths *vs.* 1.8 expected). In the older age group of 60–79 years, the SMR had fallen but remained statistically significant (males 167 (124–221), 5983 pyears; 49 deaths *vs*. 29 expected, females 179 (127–246) 7998 pyears; 21 deaths *vs.* 8 expected).

Separate analyses for CHD mortality were carried out for the period before January 1992, between January 1992–December 2008, and from 2009 to December 2015. Over the three time periods in general, SMR mortality fell in each age category as expected (Supplementary Table 3). As shown in Fig. 1C and Table 2, in males with SFH, there was significant excess coronary mortality in the first two periods, falling from an SMR of 356 (178–637) to 255 (198–232), but post 2008 CHD mortality was no longer statistically significant (159 (91–258)). By comparison in females, although the initial high rate pre 1992 fell from 498 (215–982) to 173 (117–247) in the 1992–2008 period, the SMR was high post 2008 (350 (192–588). In NSFH patients, the CHD SMRs were low at all time periods in both males and females and only reached statistical significance in males in the 1992–2008 period (183 (107–293)).

**Table 2:** Univariate and multivariate factors associated with CHD mortality in SFH *vs.* NSFH patients.

Variable		Univariate associations with CHD death		Multivariable model including all variables.		Model selected using stepwise Cox regression	
		HR (95% CI)	*p* value	HR (95% CI)	*p* value	HR (95% CI)	*p* value
Age (years)	Per 10 years	1.80 (1.59–2.04)	<0.0001	1.49 (1.22–1.82)	<0.0001	1.53 (1.27–1.85)	<0.0001
Sex	F:M	0.52 (0.38–0.70)	<0.0001	0.53 (0.35–0.79)	0.0002	0.54 (0.36–0.80)	0.002
Ever smoker	Y:N	2.67 (1.96–3.63)	<0.0001	1.99 (1.33–2.99)	0.001	2.06 (1.38–3.07)	0.0004
Diabetes	Y:N	3.48 (1.43–8.46)	0.006	1.74 (0.43–7.14)	0.439		
Total cholesterol (mmol/l)	Per SD	1.33 (1.15–1.54)	<0.0001	1.26 (1.08–1.48)	0.004	1.27 (1.09–1.49)	0.003
BMI (kg/m^2^)	Per SD	1.30 (1.13–1.50)	<0.0001	1.14 (0.91–1.42)	0.247		
SBP (mmHg)	Per SD	1.45 (1.26–1.66)	<0.0001	1.12 (0.92–1.37)	0.247		
Prior CHD	Y:N	8.98 (6.57–12.27)	<0.0001	4.34 (2.90–6.51)	<0.0001	4.47 (2.99–6.69)	0.003
Age >40 years at diagnosis	Y:N	3.01 (2.25–4.03)	<0.0001	1.46 (0.99–2.17)	0.058		
Family history early CHD in 1st degree relative	Y:N	1.96 (1.16–3.34)	0.012	1.40 (0.75–2.59)	0.290		
Diagnosis	SFH:NSFH	1.93 (1.33–2.79)	0.0005	1.31 (0.81–2.11)	0.274	1.22 (0.80–1.87)	0.360

Lipoprotein(a) measures not available.

Finally, we determined to what extent the excess SFH-associated CHD mortality risk was explained by traditional risk factors. As shown in Table 3, CHD mortality risk was independently related to age, sex, smoking, previous CHD and total cholesterol level at registration and, of these, prior CHD was associated with the largest HR. Overall, the hazard ratio for CHD in SFH *vs.* NSFH was 1.93 (1.33–2.79) *p* = 0.0005 before adjustment and 1.22 (80–187) *p* = 0.36 after adjustment for all variables in the stepwise model.

**Table 3:** Observed and expected deaths from CHD by time period in SFH compared with NSFH for patients aged 20–79 years.

	Total Pyrs^a^	From 1 January, 1980 to 31 December, 1991					Total Pyrs^a^	From 1 January, 1992 to December 2008				
		Observed	Expected	SMR	95% CI	*p*-value		Observed	Expected	SMR	95% CI	*p*-value
Severe–FH males	1742	11	3.09	356.3	(178,637)	<0.0008	13522	69	27.1	254.6	(198, 322)	<0.0001
Severe–FH females	15647	8	1.61	498.3	(215,982)	0.005	11026	30	17.3	173.1	(117,247)	0.008
Non-severe-FH males	458	1	1.58	63.4	(2353)	1	3349	17	9.3	183.1	(107,293)	0.03
Non-severe-FH females	787	2	0.86	231.7	(28,837)	0.42	7134	7	8.0	87.4	(35,180)	0.90

	Total Pyrs^a^	From 1 January, 2009 to 31 December, 2015					*p* value trend
		Observed	Expected	SMR	95% CI	*p*-value	
Severe-FH males	5771	16	10.1	158.8	(91,258)	0.10	0.03
Severe–FH females	3816	14	4.0	350.3	(192,588)	0.0001	0.68
Non-severe-FH males	1550	5	2.7	183.5	(60,428)	0.28	0.41
Non-severe-FH females	3400	3	2.1	140.8	(29,411)	0.72	0.96

a Pys = person years exposure.

## 4 Discussion

In the UK Simon Broome FH Register, 67.7% of patients fulfil the IAS definition of SFH, based on their baseline characteristics. High levels of Lp(a) and creatinine are also components of the SFH definition [6], but were not available for this cohort, and a proportion of the NSFH patients would be moved to the SFH category if this data were available, so this figure is an underestimate of the true prevalence of SFH in this cohort. The data show that, despite current treatments, CHD mortality is markedly elevated in SFH patients and supports the view that attaining optimal lipid lowering, as well as management of other risk factors, will be of clinical benefit. The data provide an evidence base for the IAS proposed categorisation [6], and for the subsequent stratified management of those with non-severe and severe-FH. The high CHD risk in treated SFH was of similar magnitude in both men and women (with an SMR of 237 and 226 respectively), but while the CHD risk was elevated in treated NSFH men (SMR of 168), this was not the case in NSFH women (SMR of 109), suggesting that CHD risk in the majority of this group is being appropriately treated.

Overall, the hazard ratio for CHD in SFH was 64% higher than in NSFH, with CHD mortality risk being independently related to age, sex, smoking, previous CHD, total cholesterol and age above 40 years and untreated at diagnosis, with the major determinant of risk being a prior diagnosis of CHD. After adjustment for these variables in a stepwise model, the mortality risk in SFH and NSFH was not significantly different, indicating that the excess risk in the SFH patients can be accounted for mainly by these risk factors. These data reinforce that early diagnosis, before CHD has occurred, is likely to be of major benefit in reducing morbidity in subjects with FH. We have previously reported that, except for low HDL-C, a range of novel and emerging CHD risk factors were not independently associated with CHD risk in this cohort [13]. Overall, the ten year CHD mortality rate for SFH at baseline was around 2-fold higher than for those with NSFH, with similar fold differences over the whole age range. The SMR for CHD was extreme in both men and women in the 20–39 year old age range, but the number of events is small and the CIs were large. In middle age, the CHD SMRs were over 350 in men and 500 in women, and although the death rate was lower in patients over 60 years of age, the SMRs in both SFH men and women remained significantly elevated. This contrasts with a “survivor” effects previously reported in the overall register data [14], where the CHD SMR was not significantly raised at this age than in the general population.

The Spanish FH Registry has recently reported that the use of the SFH/NSFH definition did not improve the ability to identify subjects at highest risk of cardiovascular disease after adjustment for traditional risk factors [15], although this analysis was based on cross-sectional CHD data and not prospective longitudinal mortality available from the UK Register. Our data confirm that the higher CHD risk in SFH *vs.* NSFH patients can be explained largely by the higher prevalence of traditional CHD risk factors in the SFH group and, as such, this definition may be useful to guide patient clinical management. The strengths of the analysis presented here is that it is based on a large dataset with essentially complete follow-up over a period of more than 20 years, with more than 57,000 person years of exposure. However, a limitation of the data is that the number of events in later periods is relatively small so the confidence intervals are large and point estimates need to be interpreted cautiously. We also accept that the NSFH category will include a significant proportion of patients with polygenic hypercholesterolaemia [16]. A more accurate assessment would be provided by an analysis restricted to patients with genetically diagnosed FH, however, this data is not available for the majority of Register patients who were recruited in the era before DNA testing was routinely available, and in clinical practice this is not yet routinely available in the UK nor in the majority of countries world-wide. However, a mutation can be found in up to 80% of patients with DFH but only 20–30% of those with PFH, most of whom have a polygenic and not a monogenic cause of their clinical phenotype [1,2]. In analysis confined to those with a diagnosis of DFH, the CHD mortality rate was 74% higher in the SFH compared to the NSFH group, while in the PFH patients, the difference was only 26% higher, supporting the view that the highest CHD mortality group will be those with a clinical characteristics of SFH who also carry an FH-causing mutation.

A limitation of the data is that we do not have current data on whether the FH patients in the cohort have been treated with statin or other lipid-lowering agents and only have their lipid levels at registration, but insights into current treatment practice can be obtained from the 2010 audit of the management of FH patients [18], which included the clinics where the patients were originally recruited. Data was available from the notes of 2324 adult patients with clinical FH; 86% were on statin treatment (33% were treated with atorvastatin, 33% with rosuvastatin, 15% with simvastatin) and 40% were additionally being treated with ezetimibe. Mean (SD) untreated LDL-C was 6.44 (1.77) mmol/l, which by the third clinic visit (at the time of audit) had been lowered to a mean of 3.60 (1.48) mmol/l, representing an overall median reduction of 47% from baseline. The remainder were taking a resin (4%), statin-intolerant (6.8%), declined statin treatment (1.9%) or were pregnant or breastfeeding (1.7%). We believe that there is a high likelihood that such treatments were also being given to the SB cohort of patients, as is recommended by all UK NICE FH and lipid management guidelines.

These data can be used to estimate what proportion of SFH patients may qualify for treatment with PCSK9 monoclonal antibody inhibitors. The NICE Technical Appraisals TA393/394 (https://www.nice.org.uk/guidance/ta393/ and https://www.nice.org.uk/guidance/ta394/ and the 2017 updated NICE FH guideline (https://www.nice.org.uk/guidance/cg71), recommend alirocumab or evolocumab if a patient has heterozygous-FH with proven CVD, and persistently high LDL-C >3.5 mmol/l despite maximal tolerated lipid-lowering therapy, or heterozygous-FH with persistently high LDL-C >5.0 mmol/l despite maximal tolerated lipid-lowering therapy [17]. In the Simon Broome Register, 25% of patients had CVD at baseline and might qualify depending on their treatment response. We do not have recent on-treatment lipid levels to examine this, but as shown in Supplementary Fig. 2, in the 2010 National Audit of FH patients [18], 47% had on-treatment LDL-C >3.5 mmol/l suggesting that a maximum of 12% of the register patients might qualify based on these criteria. The audit data also shows that 16% had on-treatment LDL-C >5.0 mmol/l suggesting that an additional 12% of the register patients might qualify based on these criteria. However, since highest CHD risk is seen in the SFH group, limiting the use of PCSK9i to those who fall into this category would reduce the number potentially eligible from 24% to around 16%. These estimates are in line with data from the Spanish FH registry, where 17% of patients would qualify for PCSK9 inhibition according to European guidelines [19].

In conclusion, we confirm that the IAS proposed definition of severe-FH identifies a group of male and female patients with a clinical diagnosis of FH that have a particularly high risk of CHD mortality, even when on statin treatment. The major contributing factor to this high risk is the presence of prior CHD, although the utility of the SFH definition is that it combines information from many different risk factors. The CHD death rate is significantly elevated in SFH patients at all ages, but especially in those in the 20–29 year old group and is still raised in those over the age of 60 years, where a “survivor” effect has previously been seen in the overall group of treated FH patients. As shown previously in this dataset, compared to men, women with SFH do not appear to have benefited from the availability of more potent statins in the most recent time period examined. While this could be an artefact due to the relatively small number of events in this later period, it is possible that these women are not being treated as rigorously as their male counterparts. This possibility is supported by recent reports indicating that women are undertreated with statins [20,21]. Overall, our data suggest that around 16% of patients with a definition of SFH may qualify for treatment with anti PCSK9 agents, based on current UK guidelines.

## Conflicts of interest

HAWN, NC, PND, BJ, IFWM, and HS have served as consultants to pharmaceutical companies marketing lipid-lowering drugs, and/or have received travel expenses, payment for speaking at meetings and funding for research from some of these companies. The other authors have nothing to declare.

## References

[1] Humphries S.E., Cranston T., Allen M. (2006). Mutational analysis in UK patients with a clinical diagnosis of familial hypercholesterolaemia: relationship with plasma lipid traits, heart disease risk and utility in relative tracing. J. Mol. Med..

[2] Nordestgaard B.G., Chapman M.J., Humphries S.E. (2013). Familial hypercholesterolaemia is underdiagnosed and undertreated in the general population: guidance for clinicians to prevent coronary heart disease: consensus statement of the European Atherosclerosis Society. Eur. Heart J..

[3] Slack J. (1969). Risks of ischaemic heart-disease in familial hyperlipoproteinaemic states. Lancet.

[4] Stone N.J., Levy R.I., Fredrickson D.S. (1974). Coronary artery disease in 116 kindred with familial type II hyperlipoproteinemia. Circulation.

[5] Gagne C., Moorjani S., Brun D. (1979). Heterozygous familial hypercholesterolemia. Relationship between plasma lipids, lipoproteins, clinical manifestations and ischaemic heart disease in men and women. Atherosclerosis.

[6] Santos R.D., Gidding S.S., Hegele R.A. (2016). Defining severe familial hypercholesterolaemia and the implications for clinical management: a consensus statement from the International Atherosclerosis Society Severe Familial Hypercholesterolemia Panel. Lancet Diabetes Endocrinol..

[7] Scientific Steering Committee on behalf of Simon Broome Familial Hyperlipidaemia Register Group (1991). The risk of fatal coronary heart disease in familial hypercholesterolaemia. B M J.

[8] Betteridge D.J., Broome K., Durrington P.N. (1999). Scientific Steering Committee on behalf of the Simon Broome Register Group - mortality in treated heterozygous familial hypercholesterolaemia: implications for clinical management. Atherosclerosis.

[9] Neil H.A., Hawkins M.M., Durrington P.N. (2005). Non-coronary heart disease mortality and risk of fatal cancer in patients with treated heterozygous familial hypercholesterolaemia: a prospective registry study. Atherosclerosis.

[10] Humphries S.E., Cooper J.A., Seed M. (2018). Coronary heart disease mortality in treated familial hypercholesterolaemia: update of the UK Simon Broome FH register. Atherosclerosis.

[11] Friedewald W.T., Levy R.I., Fredrickson D.S. (1972). Estimation of the concentration of low-density lipoprotein cholesterol in plasma, without use of the preparative ultracentrifuge. Clin. Chem..

[12] Coleman M., Douglas A., Hermon C. (1986). Cohort study analysis with a FORTRAN computer program. Int. J. Epidemiol..

[13] Neil H.A.W., Seagroatt V., Betteridge D.J. (2004). Risk factors for coronary heart disease in patients with heterozygous familial hypercholesterolaemia. Heart.

[14] Neil A., Cooper J., Betteridge J. (2008). Reductions in all-cause, cancer, and coronary mortality in statin-treated patients with heterozygous familial hypercholesterolaemia: a prospective registry study. Eur. Heart J..

[15] Perez-Calahorra S., Sanchez-Hernandez R.M., Plana N. (2017). Value of the definition of severe familial hypercholesterolemia for stratification of heterozygous patients. Am. J. Cardiol..

[16] Talmud P.J., Shah S., Whittall R. (2013). Use of low-density lipoprotein cholesterol gene score to distinguish patients with polygenic and monogenic familial hypercholesterolaemia: a case-control study. Lancet.

[17] Carroll C., Tappenden P., Rafia R. (2017). Evolocumab for treating primary hypercholesterolaemia and mixed dyslipidaemia: an evidence review group perspective of a NICE single technology appraisal. Pharmacoeconomics.

[18] Seed M., Roughton M., Pedersen K. (2012). Current statin treatment, DNA testing and cascade testing of UK patients with familial hypercholesterolaemia (FH): results from the Royal College of Physicians UK National audit. Primary Care Cardiovasc. J..

[19] Masana L., Plana N., Perez-Calahorra S. (2017). How many familial hypercholesterolemia patients are eligible for PCSK9 inhibition?. Atherosclerosis.

[20] Amrock S.M., Duell P.B., Knickelbine T. (2017). Health disparities among adult patients with a phenotypic diagnosis of familial hypercholesterolemia in the CASCADE-FH patient registry. Atherosclerosis.

[21] O'Keeffe A.G., Nazareth I., Petersen I. (2016). Time trends in the prescription of statins for the primary prevention of cardiovascular disease in the United Kingdom: a cohort study using the Health Improvement Network primary care data. Clin. Epidemiol..

