# Supplementary material for: Coronary heart disease mortality in severe vs. non-severe familial hypercholesterolaemia in the Simon Broome Register
Source: Atherosclerosis. 2019 Feb;281:207–12. doi: 10.1016/j.atherosclerosis.2018.11.014 (PMC6403443; doi:10.1016/j.atherosclerosis.2018.11.014)
Supplement: Supplementary materials [file mmc1.docx]

**Supplementary Figures**

**S Figure 1. SMR CHD Deaths for SFH and NSFH male and female patients (20-79 years)**

**
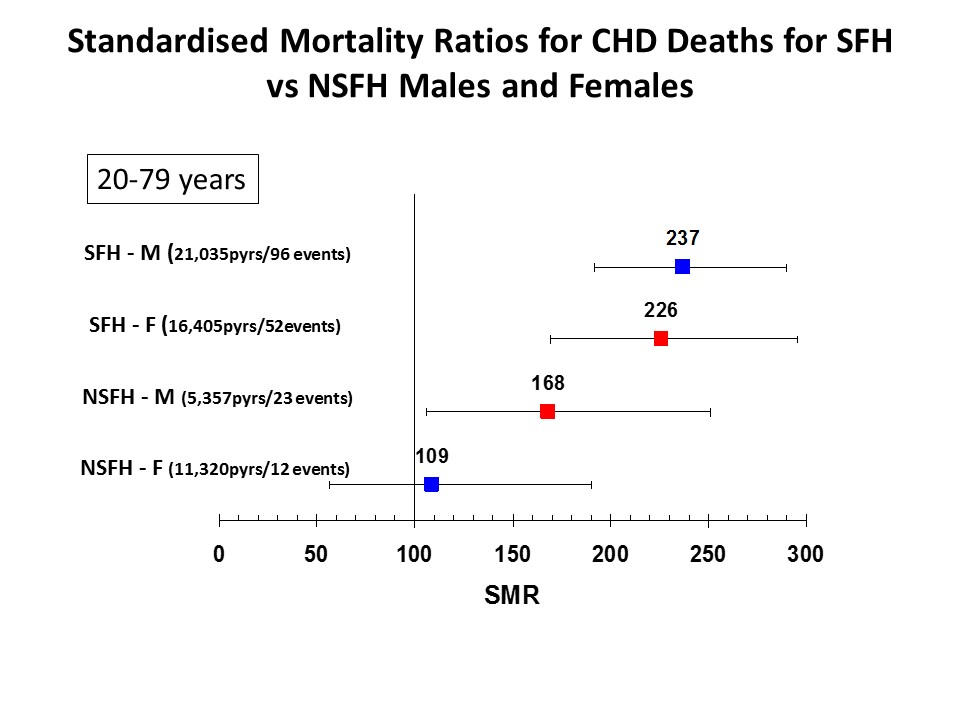
**

**Supplementary Figure 2. Histogram showing distribution of on-treatment LDL-C in 2045 FH patients with audited notes from the 2010 UK National Audit (16)**

**
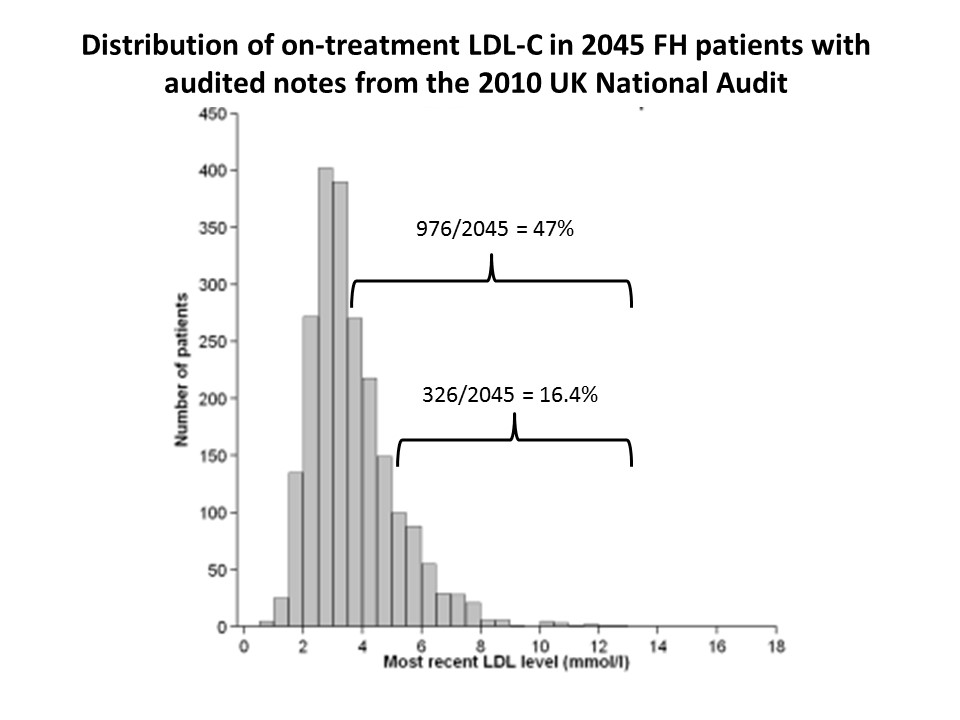
**

**Supplementary Table 1 – Distribution of High Risk factors in Simon Broome FH patients with different registration LDL-C**

| **Number of High risk factors** | **LDL > 10mmol/l**  **N = 201 (8.3%)** | **LDL <10 - >8.0mmol/l**  **N= 423 (17.4%)** | **LDL <8 - >5.0mmol/l**  **N=1809 (74.3%)** |
| --- | --- | --- | --- |
| **Mean (SD) LDL-C** | **11.4 (1.4)** | **8.8 (0.5)** | **6.4 (0.8)** |
| **0** | **2 (1%)** | 14 (3.3%) | 66 (3.6%) |
| **1** | **42 (20.9%)** | **79 (18.7%)** | 371 (20.5%) |
| **2** | **68 (33.8%)** | **153 (36.2%)** | **653 (36.1%)** |
| **3** | **56 (27.9%)** | **127 (30.0%)** | **463 (25.6%)** |
| **4** | **27 (13.4%)** | **42 (9.9%)** | **212 (11.7%)** |
| **>4** | **6 (3.0%)** | **8 (1.9%)** | **44 (2.4%)** |
| **Number (%age) of SFH** | **201 (100%)** | **409 (96.6%)** | **1372 (75.8%)** |

Note : of the original 2942, 496 had LDL-C below 5.0mmol/l but above 4.99mmol/l

Difference in distribution of risk factors Chi Square p = 0.21

**Supplementary Table 2. CHD SMR in Simon Broome DFH vs PFH patients with SFH and NSFH (all ages post 1991)**

|  | **Total pyears** | **Observed events** | **Predicted events** | **SMR** | **Lower CI** | **Upper CI** | **P value** |
| --- | --- | --- | --- | --- | --- | --- | --- |
| **DFH - SFH** | 19004 | 80 | 31.3 | 255* | 202 | 318 | 5.4 x 10 ^-13^ |
| **PFH - SFH** | 15130 | 49 | 27.4 | 179 | 133 | 237 | 0.0002 |
| **DFH - NSFH** | 7915 | 15 | 10.3 | 146 | 81 | 240 | 0.20 |
| **PFH - NSFH** | 7518 | 17 | 12.0 | 142 | 83 | 227 | 0.20 |

*DFH-SFH PFH-SFH p = 0.05

**Supplementary Table 3 – Observed and expected deaths from CHD by age group and time period for SFH patients**

|  | **1 January 1980 to 31 December 1991** | | | | | | | **1 January 1992 to 31 December 2008** | | | | | | |
| --- | --- | --- | --- | --- | --- | --- | --- | --- | --- | --- | --- | --- | --- | --- |
| **Attained age (years)** | **Person years observation** | **Observed** | **Expected** | **SMR** | **95% CI** | **p-value** | **Rate/ 100000** | **Person years observation** | **Observed** | **Expected** | **SMR** | **95%CI** | **p-value** | **Rate/ 100000** |
| 20-39 | 1054 | 5 | 0.07 | 7392 | (240,17251) | 2.2 x10^-08^ | 474 | 4774 | 4 | 0.22 | 1782 | (486,4563) | 0.0001 | 84 |
| 40-59 | 1754 | 11 | 1.97 | 560 | (279,1001) | 1.4 x10^-05^ | 627 | 11168 | 38 | 8.78 | 433 | (306,594) | 5.4 x10^-13^ | 340 |
| 60-79 | 498 | 3 | 2.66 | 113 | (23,330) | 0.99 | 602 | 8605 | 57 | 35.42 | 161 | (122,721) | 0.001 | 662 |
| 0-79 | 3306 | 19 | 4.69 | 405 | (244, 632) | 1.1 x 10^-06^ | 575 | 24548 | 99 | 44.42 | 223 | (181,271) | 2.55 x 10^-12^ | 403 |

|  | **1 January 2009 to 31 December 2015** | | | | | | |
| --- | --- | --- | --- | --- | --- | --- | --- |
| **Attained age (years)** | **Person years observation** | **Observed** | **Expected** | **SMR** | **95% CI** | **p-value** | **Rate/ 100000** |
| 20-39 | 953 | 1 | 0.03 | 3273 | (83,18,234) | 0.067 | 105 |
| 40-59 | 3756 | 2 | 1.76 | 113 | (14,408) | 1 | 53 |
| 60-79 | 4878 | 27 | 12.27 | 220 | (145,320) | 0.0004 | 554 |
| 0-79 | 9587 | 30 | 14.07 | 213 | (144,304) | 0.0003 | 313 |
